# Sex-specific association between the cortisol awakening response and obsessive-compulsive symptoms in healthy individuals

**DOI:** 10.1186/s13293-019-0273-3

**Published:** 2019-12-02

**Authors:** Cristian Sebastian Melia, Virginia Soria, Neus Salvat-Pujol, Ángel Cabezas, Roser Nadal, Mikel Urretavizcaya, Alfonso Gutiérrez-Zotes, José Antonio Monreal, José Manuel Crespo, Pino Alonso, Elisabet Vilella, Diego Palao, José Manuel Menchón, Javier Labad

**Affiliations:** 1 Department of Psychiatry, Bellvitge University Hospital, Bellvitge Biomedical Research Institute (IDIBELL), Neurosciences Group—Psychiatry and Mental Health, Barcelona, Spain; 2 Centro de Investigación Biomédica en Red de Salud Mental (CIBERSAM), Carlos III Health Institute, Madrid, Spain; 3 Department of Clinical Sciences, School of Medicine, Universitat de Barcelona, Barcelona, Spain; 4 Hospital Universitari Institut Pere Mata, IISPV, Universitat Rovira i Virgili, Reus, Spain; 5 Institut de Neurociències, Universitat Autònoma de Barcelona, Cerdanyola del Vallès, Spain; 6 Department of Mental Health, Parc Taulí Hospital Universitari, I3PT, Universitat Autònoma de Barcelona, Sabadell, Spain

**Keywords:** Cortisol, Stress, Obsessive, OCD, Sex differences

## Abstract

**Background:**

Previous studies have shown associations between obsessive-compulsive disorder (OCD) and hypothalamic-pituitary-adrenal axis activity (HPA). We aimed to investigate the association between obsessive-compulsive (OC) symptoms and HPA axis functionality in a non-clinical sample and to explore whether there are sex differences in this relationship.

**Methods:**

One hundred eighty-three healthy individuals without any psychiatric diagnosis (80 men, 103 women; mean age 41.3 ± 17.9 years) were recruited from the general population. The Obsessive-Compulsive Inventory Revised (OCI-R) was used to assess OC symptoms. State-trait anxiety, perceived stress, and stressful life events were also assessed. Saliva cortisol levels were determined at 6 time points (awakening, 30 and 60 min post-awakening, 10:00 a.m., 23:00 p.m. and 10:00 a.m. the following day of 0.25 mg dexamethasone intake [that occurred at 23:00 p.m.]). Three HPA axis measures were calculated: cortisol awakening response (CAR), cortisol diurnal slope, and cortisol suppression ratio after dexamethasone (DSTR). Multiple linear regression analyses were used to explore the association between OC symptoms and HPA axis measures while adjusting for covariates. Our main analyses were focused on OCI-R total score, but we also explored associations with specific OC symptom dimensions.

**Results:**

No significant differences were observed between males and females in OC symptoms, anxiety measures, stress, or cortisol measures. In the multiple linear regression analyses between overall OC symptoms and HPA axis measures, a female sex by OC symptoms significant interaction (standardized beta = − 0.322; *p* = 0.023) for the CAR (but not cortisol diurnal slope nor DSTR) was found. Regarding specific symptom dimensions, two other sex interactions were found: a blunted CAR was associated with obsessing symptoms in women, whereas a more flattened diurnal cortisol slope was associated with ordering symptoms in men.

**Conclusions:**

There are sex differences in the association between OC symptoms and HPA axis measures in healthy individuals.

## Introduction

Obsessive-compulsive disorder (OCD) is a common chronic long-lasting psychiatric disorder, with a prevalence of 2.3% that interferes with all aspects of life of the individual [1]. OCD is characterized by the presence of obsessions, repetitive disturbing and uncontrollable thoughts that persist despite the patient’s efforts to suppress or ignore them, and compulsions and repetitive and ritualized behaviors or mental acts aimed at neutralizing obsession-induced anxiety [2]. Obsessive-compulsive (OC) symptoms are not exclusive of OCD. They are prevalent in non-clinical samples, with clinically relevant symptoms in about 21.7% of the general population [3]. The vulnerability to compulsive activity can be predicted by a spectrum of neuropsychological mechanisms such as impaired motor inhibition, cognitive inflexibility and an imbalance in goal-directed vs habit learning [4]. A series of cortico-striato-thalamo-cortical circuits that are associated with these cognitive changes are believed to underpin the expression of compulsive behaviors [4].

The hypothalamic-pituitary-adrenal (HPA) axis, the major stress response system of the body, is known to be involved in the susceptibility to develop psychiatric disorders and physical conditions such as infectious diseases, cardiovascular problems, autoimmune processes, chronic fatigue syndrome, and rheumatoid arthritis [5]. Stressful life events and dysregulations of the HPA axis are thought to play a role in the pathogenesis of the OCD [6, 7] and other anxiety disorders [8]. The first studies to explore the relationship between HPA axis functionality and OCD measured HPA hormones in cerebrospinal fluid (CSF) and blood. For instance, elevated values of corticotropin releasing hormone (CRH) in CSF [9], increased nocturnal secretion of adrenocorticotropic hormone (ACTH) [10], and higher basal serum cortisol values [11, 12] have been described in OCD patients.

In the last two decades, the study of HPA axis functionality using saliva samples has attracted great interest [13]. Saliva collection allows cortisol determination at different moments of the day with a non-invasive procedure. Therefore, saliva sampling can be used for studying several dynamic tests of the HPA axis, such as the cortisol awakening response (CAR), the diurnal cortisol rhythm, or the negative feedback of the HPA axis with the study of the cortisol suppression by dexamethasone. We have used this approach to study the role of these HPA axis measures in patients with OCD, major depressive disorder (MDD) and healthy individuals [6]. A more flattened diurnal cortisol slope calculated between 10 and 23 h was observed for OCD patients with comorbid MDD. We also found that trait anxiety was a moderator of the relationship between OCD and HPA axis measures, as OCD patients with greater trait anxiety showed an increased CAR and reduced cortisol suppression after dexamethasone administration. Saliva sampling also allows the study of the cortisol response to psychosocial stress. In this line, previous studies exploring the cortisol response to the Trier Social Stress Test, a social stress procedure, in patients with OC personality disorder have revealed an attenuated response in male patients compared to male controls, whereas no significant differences were found in women [14].

There is a wealth of data describing sex differences in HPA axis, documented as early as the neonatal period and at all individual levels of the HPA axis [15]. Evidence on sex differences in the HPA axis response to stress is conflicting, with some studies showing no differences. But the overall picture seems to indicate that the response to psychological stress in adult men is with greater increases in cortisol in comparison to women [5]. However, the study of sex differences in the HPA axis response is more complex if we consider potential moderating variables. For instance, the type of the stressor seems to influence the HPA axis response: men show greater cortisol responses to a mathematical and verbal challenge whereas women show greater cortisol responses to a social rejection challenge [16]. Age and gender are also moderators of the HPA response to psychosocial stress. In a study exploring ACTH and cortisol responses to the TSST in healthy elderly adults, younger adults, and children, there was no age effect in the subgroup of women, whereas younger men had higher ACTH responses compared to older men [17]. In relation to free salivary cortisol, an enhanced cortisol response in elderly men compared to elderly women was observed, while no gender differences emerged in neither young adults nor children [17].

The study of potential sex differences regarding HPA axis activity in the field of OCD has been an underresearched area. This approach is important because sex plays a role in the clinical expression of the illness: women show, when compared to men, more contamination/cleaning symptoms [18–21], more aggressive obsessions and hoarding symptoms [20], more somatic obsessions [19], less sexual/religious obsessions [18, 22], and less checking and repeating compulsions [22]. Conversely, other studies have failed to find sex differences in OCD symptom dimensions [23]. However, in that study correlations between the distinct types of OCD symptom dimensions were stronger in men compared to women. It is plausible that sex differences in HPA axis activity could contribute to the different clinical expression of obsessive-compulsive symptoms between men and women. However, previous studies studying HPA axis measures in OCD [6, 9–12] did not specifically explore whether there were sex differences on these relationships.

The main aim of our study was to explore the role of the HPA axis in the clinical expression of obsessive-compulsive (OC) symptoms in a non-clinical sample. To our knowledge, this issue has not been studied up to date. As there are sex differences in the presentation of OC symptoms and HPA axis activity, we also aimed to explore whether there are sex differences in this relationship.

## Methods

### Study sample

One hundred eighty-three healthy individuals (80 men, 103 women; mean age 41.3 ± 17.9 years) were recruited from the general population by advertisements in the community. Recruitment was conducted in two provinces (Barcelona, Tarragona) from Catalonia (Spain) by clinical researchers from the Department of Psychiatry at Bellvitge University Hospital (Hospitalet de Llobregat, Barcelona) and from the Hospital Universitari Institut Pere Mata (Reus, Tarragona). All participants had no past or current history of psychiatric disorders (including OCD) and a score below 7 on the 28-item Spanish adaptation of the Goldberg General Health Questionnaire (GHQ-28) [24]. Exclusion criteria were age less than 18 years, a diagnosis of a psychiatric disorder including substance abuse or dependence (except nicotine), mental retardation, neurological disorders, severe medical conditions, pregnancy, or puerperium and corticosteroid treatment in the previous 3 months. Two women were receiving contraceptive pills. The research protocol was approved by the Ethics Committees of Bellvitge University Hospital and Hospital Universitari Sant Joan, and all participants provided written informed consent after having received a full explanation of the study.

### Clinical assessment

From a semi-structured interview, a sort of sociodemographic and clinical variables and substance use were assessed. In order to calculate the body mass index, weight and height were measured in all participants, using the formula (kg)/height(m^2^).

The assessment of the OC symptoms was performed using the Obsessive-Compulsive Inventory Revised (OCI-R). The OCI-R consists of a self-reported test of 18 items divided on six subscales: washing, checking, ordering, obsessing, hoarding, and neutralizing. Each item is scored on a 5-point scale (0–4 points), and the total score is the sum of the scores on all items. It also provides scores for the six subscales [25]. The OCI-R has proved its validity in both clinical [25, 26] and non-clinical samples [27] and has been validated to Spanish [28]. A cut-off score of 21 has been recommended, with scores at or above this level indicating the likely presence of OCD [25].

Several psychometric scales were administered to assess anxiety and stress measures of all participants. Current and trait anxiety were measured with the State-Trait Anxiety Inventory [29]. Stressful life events that occurred in the 6 previous months were assessed with the Holmes-Rahe Social Readjustament Scale [30], that has been also used in Spanish populations [31]. Perceived stress during the previous month was assessed with the 14-item Perceived Stress Scale (PSS) [32].

### Collection of saliva samples

Saliva samples were obtained using Salivette (Sarstedt AG & Co., Nümbrecht, Germany) containers. Participants were instructed to collect repeated saliva samples at home during a regular day and to avoid intense physical activity and stressful situations. Fifteen minutes before sample collection, participants were told not to eat, drink, smoke, or brush their teeth.

Saliva samples were obtained at awakening (T1), 30 (T2) and 60 (T3) min after awakening, at 10 a.m. (T4) and at 11 p.m. (T5) on the same day. Participants were told to take a very low dose of dexamethasone (0.25 mg) at 11 p.m. just after T5 sampling. Another saliva sample was obtained the day after at 10 a.m. (T6).

The samples were kept refrigerated and returned personally by each participant. After receipt, the Salivettes were stored at − 20 °C and sent to the BioBank from the Institut de Investigació Sanitaria Pere Virgili (IISPV) for centrifugation (3000 rpm for 5 min) and aliquotation, then frozen at − 20 °C until analysis.

### Cortisol measurements

An enzyme-linked immunosorbent assay (ELISA) was performed to determine the cortisol levels in the saliva samples (IBL International, Hamburg, Germany). The intra-assay and inter-assay coefficients of variation were under 8%. The sensitivity of the assay was 0.08 nmol/L. To assess the HPA axis function, three dynamic tests were used: the Cortisol Awakening Response (CAR), the slope between morning and evening cortisol, and the Dexamethasone Suppression Test Ratio (DSTR) with very low doses of dexamethasone (0.25 mg).

The CAR is a physiological process, consisting in the increase of cortisol levels as a response to awakening in the morning [33]. It combines features of a reactivity index (response to awakening) with aspects tied to circadian regulation and it has been related to a wide range of psychosocial, physical, and mental health parameters [13]. The CAR was calculated, as suggested by Pruessner and colleagues, using the area under the curve with respect to the increase [34]. The calculation included the sampling points of T1-T3 samples.

The diurnal cortisol slope is defined as the rate of decline in cortisol levels during the day, from morning to evening. It was calculated using the T4 and the T5 samples. Previous studies have calculated the diurnal cortisol slope using both awakening to bedtime or fixed time points (morning to evening), and the implications of this choice has received little attention [35]. In a previous study by our group, we detected significant differences between OCD patients with or without major depression in a fixed time points diurnal cortisol slope (calculated between 10 a.m. and 11 p.m.) but not in the awakening to evening diurnal cortisol slope [6]. Some authors have suggested that the CAR is influenced by different biological mechanisms than the rest of the diurnal cortisol rhythm [36]. For this reason, in this study, we have decided to use the diurnal cortisol slope using fixed time points that do not include the awakening response (between 10 a.m. and 11 p.m.).

The DSTR reflects the cortisol suppression ratio to dexamethasone, a glucocorticoid receptor agonist and provides information about negative feedback of the HPA axis. Thus, a lack of suppression after dexamethasone administration is considered a measure of glucocorticoid resistance. The DSTR was defined as the ratio of cortisol between T4 and T6 samples (equivalent to the ratio between cortisol at 10 am of two consecutive days: before and after dexamethasone administration). Higher ratios were indicative of greater suppression after dexamethasone administration.

### Statistical analysis

Data processing was performed using SPSS 23.0 (SPSS, IBM, USA). Normal distribution for all variables was explored using histograms and normality tests (Kolmogorov-Smirnov). In order to test for deviations from normality, the distribution of all continuous variables was explored. Measures with a skewed distribution were log transformed (ln) before its use in parametric tests (e.g., *T* test, Pearson correlations) or in linear regression analyses when used as dependent variables. This was the case for OCI-R and Holmes-Rahe Social Readjustment scales. However, for cortisol values, we opted for a power transformation (*X*’ = (*X*^0.26^ − 1)/0.26) to normalize the data, as proposed by Miller and Plessow [37]. The diurnal cortisol slope was calculated with and without transformed cortisol concentrations. However, when parametric tests and multivariate analyses were performed, slopes were calculated using transformed cortisol values. To calculate the DSTR, untransformed cortisol values were used, performing the power transformation after calculating the ratio [38].

*T* tests were used to compare continuous data between men and women. Pearson correlations were used to explore the relationship between continuous variables. Significance was set at *p* < 0.05 (bilateral).

To explore the relationship between the status of the HPA axis, the OC symptoms, and the psychometric scales, we conducted multiple regression analyses, considering the HPA axis measures as the dependent variable in each case. The following independent variables were entered in each model with the enter procedure: female gender, age, BMI, smoking, OCI-R scores, Holmes-Rahe Social Readjustament Scale stress score, STAI-trait score, and STAI-state anxiety score. The decision for including these variables was based on the fact that they are known moderators of HPA axis activity [5, 6, 13, 39]. As PSS showed a high correlation with STAI anxiety subscales, we opted for not including this variable in the multiple linear regression analyses. Potential sex by OCI-R interactions were tested with a forward selection procedure. Therefore, only significant interactions were included in the final equations.

First, we performed three separate multiple regression analysis using, in each one, a different HPA axis measure (CAR, diurnal cortisol slope, and DSTR) as the dependent variable. In these models, the OC symptoms, as the total score of the OCI-R, together with stressful life events and anxiety measures, were considered the independent variables.

We also performed additional multiple regression analyses as an exploratory approach considering different subscales of the OCI-R (checking, hoarding, neutralizing, obsessing, ordering, and washing) in relation to each HPA axis measure. In these multiple linear regression analyses, one equation was performed for each OCI-R subscore that was included in the model as an independent variable, along with the same covariates as described before. Potential sex by OCI-R subscores interactions were also tested.

As only two women were receiving oral contraceptive pills, we did not control for this treatment in multivariate analyses. However, we repeated the main analyses after excluding these two participants to be sure that the results did not change.

Sample size calculation was performed with G power 3.1.9.2. (Franz Faul, Universität Kiel, Germany). With an alpha error of 0.05 and a beta error of 0.20 (statistical power of 80%), considering an effect size (*f*^2^) of 0.1 (small to medium), the required sample size was determined to be 172 for testing the main hypothesis with multiple linear regression analyses that included 10 predictors.

## Results

### Characteristics of the sample

Demographic and clinical characteristics of the sample are shown in Table 1. Significant sex differences were found in BMI, as men had higher BMI than women. No significant sex differences were found regarding age, OCI-R total score and the OCI-R subscales and the different psychometric tests evaluated. There were no sex differences in cortisol concentrations at the different sampling points or the three derived HPA axis measures (Table 2).

**Table 1:** Clinical characteristics from the sample

	Men (*N* = 80)	Women (*N* = 103)	*p*
Age (years)	41.4 (18.4)	41.3 (17.6)	0.969
Education (years)	12.8 (3.7)	13.5 (3.8)	0.207
BMI (kg/m^2^)	26.0 (4.6)	24.0 (4.7)	0.006
Smokers, *n* (%)	19 (23.8)	16 (15.5)	0.161
Tobacco consumption (cig/d), all participants	3.3 (8.1)	1.4 (4.2)	0.058
Tobacco consumption (cig/d), only smokers	14.2 (11.4)	9.3 (6.6)	0.144
OCI-R			
OCI-R total score	9.8 (8.8)	9.7 (8.9)	0.895
OCI-R hoarding score	2.4 (2.2)	2.7 (2.6)	0.823
OCI-R checking score	1.6 (1.8)	1.4 (2.1)	0.103
OCI-R ordering score	3.0 (2.5)	3.0 (2.1)	0.547
OCI-R neutralizing score	0.7 (1.5)	0.6 (1.2)	0.616
OCI-R washing score	0.6 (1.4)	0.7 (1.3)	0.254
OCI-R obsessing score	1.5 (2.2)	1.4 (1.9)	0.972
OCI-R ≥ 21, *n* (%)	10 (12.5)	12 (11.7)	0.861
STAI			
STAI-Trait Anxiety subscore	13.0 (8.0)	14.9 (8.7)	0.131
STAI-State Anxiety subscore	11.7 (7.9)	10.9 (6.7)	0.462
Perceived stress scale	17.0 (7.2)	17.7 (7.5)	0.523
Holmes-Rahe Social Readjustment Scale			
Number of stressful life events	2.8 (2.6)	3.0 (2.6)	0.572
Stress Score	82.3 (8.8)	90.1 (80.3)	0.356

Abbreviations: *BMI* body mass index, *cig/d* cigarettes per day, *OCI-R* Obsessive-Compulsive Inventory—Revised, *STAI* State-Trait Anxiety Inventory. Quantitative variables are presented as mean and standard deviation (SD).

**Table 2:** HPA axis measures by sex

	Men *N* = 80	Women *N* = 103	*p*
Awakening cortisol (T1), nmol/L	16.0 (11.7)	17.2 (9.0)	0.256
30-min post-awakening cortisol (T2), nmol/L	25.0 (12.6)	24.6 (14.1)	0.499
60-min post-awakening cortisol (T3), nmol/L	19.9 (12.5)	21.3 (12.6)	0.559
10 a.m. cortisol (T4), nmol/L	13.4 (12.2)	12.1 (8.3)	0.780
11 p.m. cortisol (T5), nmol/L	3.7 (5.6)	3.1 (2.3)	0.965
10 a.m. post-dexamethasone cortisol (T6), nmol/L	7.7 (5.6)	3.1 (2.3)	0.229
CAR^†^	35.8 (59.8)	26.0 (61.6)	0.295
Diurnal cortisol slope^‡^	− 0.95 (0.69)	− 1.08 (0.71)	0.393
DSTR^§^	4.7 (7.1)	10.3 (20.4)	0.086

Abbreviations: *CAR* cortisol awakening response, *DSTR* dexamethasone suppression test ratio

All variables presented in mean (SD). For cortisol concentrations (T1-T6), diurnal cortisol slope and DSTR, raw data are shown. *P* values for these HPA axis measures are generated with transformed cortisol values (in the transformation of the DSTR, we calculated the ratio with untransformed cortisol values and performed the power transformation after calculating the ratio)

^†^CAR was calculated with transformed cortisol values

^‡^Diurnal cortisol slope was calculated using 10 a.m. and 11 p.m. samples

^§^DSTR = 10 a.m. cortisol/10 a.m. post-dexamethasone cortisol

### Correlation analyses

We explored the relationship of cortisol measurements with the OCI-R and the psychometric tests. Taking all data together, the CAR correlated significantly with stressful life events (*r* = 0.17, *p* = 0.021), but not with perceived stress or trait/state anxiety. When stratifying by sex (Table 3), CAR correlated with the number of stressful life events (*r* = 0.30; *p* = 0.010) and Holmes-Rahe stress score (*r* = 0.29, *p* = 0.021) only in males.

**Table 3:** Sex-stratified correlation analyses exploring the association between psychometric scales and hypothalamic-pituitary-adrenal axis measures

Men (*N* = 80)								
	Number of SLEs	HRSRS stress score	PSS total score	STAI-state anxiety	STAI-trait anxiety	CAR	Diurnal cortisol slope	DSTR
Number of SLEs	1	0.835^**^	0.163	0.109	0.080	0.300^**^	0.210	− 0.133
HRSRS stress score	0.835^**^	1	0.073	− 0.001	− 0.059	0.290^*^	0.225	− 0.147
PSS total score	0.163	0.073	1	0.595^**^	0.603^**^	0.133	0.202	− 0.145
STAI-state anxiety	0.109	− 0.001	0.595^**^	1	0.624^**^	− 0.091	0.012	− 0.066
STAI-trait anxiety	0.080	− 0.059	0.603^**^	0.624^**^	1	0.129	0.219	0.164
CAR	0.300^**^	0.290^*^	0.133	− 0.091	0.129	1	0.332^**^	− 0.064
Diurnal cortisol slope	0.210	0.225	0.202	0.012	0.219	0.332^**^	1	0.043
DSTR	− 0.133	− 0.147	− 0.145	− 0.066	0.164	− 0.064	0.043	1
OCI-R total score	0.180	−0.031	0.238^*^	0.235^*^	0.445^**^	0.110	0.185	0.019
OCI-R washing	0.242^*^	− 0.012	0.298^**^	0.310^**^	0.406^**^	0.207	0.241^*^	− 0.063
OCI-R checking	0.073	− 0.140	0.352^**^	0.329^**^	0.464^**^	0.118	0.004	0.037
OCI-R ordering	0.162	− 0.022	0.153	0.194	0.352^**^	0.062	0.135	0.056
OCI-R obsessing	0.070	− 0.023	0.235^*^	0.246^*^	0.402^**^	0.171	0.163	− 0.046
OCI-R hoarding	0.146	0.035	0.060	0.015	0.164	0.151	0.255^*^	− 0.186
OCI-R neutralizing	0.292^**^	0.045	0.207	0.232^*^	0.381^**^	0.105	0.149	− 0.039
Women (*N* = 103)								
	Number of SLEs	HRSRS stress score	PSS total score	STAI-state anxiety	STAI-trait anxiety	CAR	Diurnal cortisol slope	DSTR
Number of SLEs	1	0.832^**^	0.120	− 0.184	− 0.003	0.091	0.045	− 0.078
HRSRS stress score	0.832^**^	1	0.248^*^	0.016	0.254^*^	0.092	0.080	− 0.013
PSS total score	0.120	0.248^*^	1	0.495^**^	0.630^**^	− 0.067	0.051	0.074
STAI-state anxiety	− 0.184	0.016	0.495^**^	1	0.611^**^	0.013	− 0.058	0.088
STAI-trait anxiety	− 0.003	0.254^*^	0.630^**^	0.611^**^	1	− 0.147	− 0.017	0.130
CAR	0.091	0.092	− 0.067	0.013	− 0.147	1	0.381^**^	0.148
Diurnal cortisol slope	0.045	0.080	0.051	− 0.058	− 0.017	0.381^**^	1	0.151
DSTR	− 0.078	− 0.013	0.074	0.088	0.130	0.148	0.151	1
OCI-R total score	0.128	0.270^*^	0.354^**^	0.131	0.470^**^	− 0.176	− 0.013	− 0.036
OCI-R washing	0.066	0.085	0.100	0.019	0.224^*^	0.019	0.043	0.057
OCI-R checking	0.042	0.163	0.239^*^	− 0.069	0.323^**^	− 0.120	− 0.031	0.046
OCI-R ordering	0.087	0.277^**^	0.311^**^	0.185	0.352^**^	− 0.220^*^	− 0.046	− 0.127
OCI-R obsessing	− 0.085	0.005	0.455^**^	0.224^*^	0.561^**^	− 0.196	0.110	0.095
OCI-R hoarding	0.232^*^	0.298^**^	0.168	− 0.014	0.232^*^	0.001	0.021	− 0.032
OCI-R neutralizing	0.065	0.238^*^	0.406^**^	0.145	0.459^**^	− 0.138	0.007	− 0.019

Pearson’s correlation coefficients are shown. Significant results are marked with an asterisk (^*^p < 0.05; ^**^p < 0.01)

Abbreviations: *SLEs* stressful life events, *HRSRS* Holmes-Rahe Social Readjustment Scale, *STAI* State-Trait Anxiety Inventory, *CAR* cortisol awakening response, *DSTR* dexamethasone suppression test ratio, *OCI-R* Obsessive-Compulsive Inventory—Revised.

No correlation was found between OC symptoms and HPA axis measures in the whole sample, but, stratifying by sex, a blunted CAR was associated with ordering symptoms (*r* = − 0.22, *p* = 0.032) in women, whereas a more flattened diurnal cortisol slope was associated with hoarding (*r* = 0.26; *p* = 0.024) and washing (*r* = 0.24; *p* = 0.033) symptoms in men.

### Multiple linear regression analyses

**Fig. 1:**
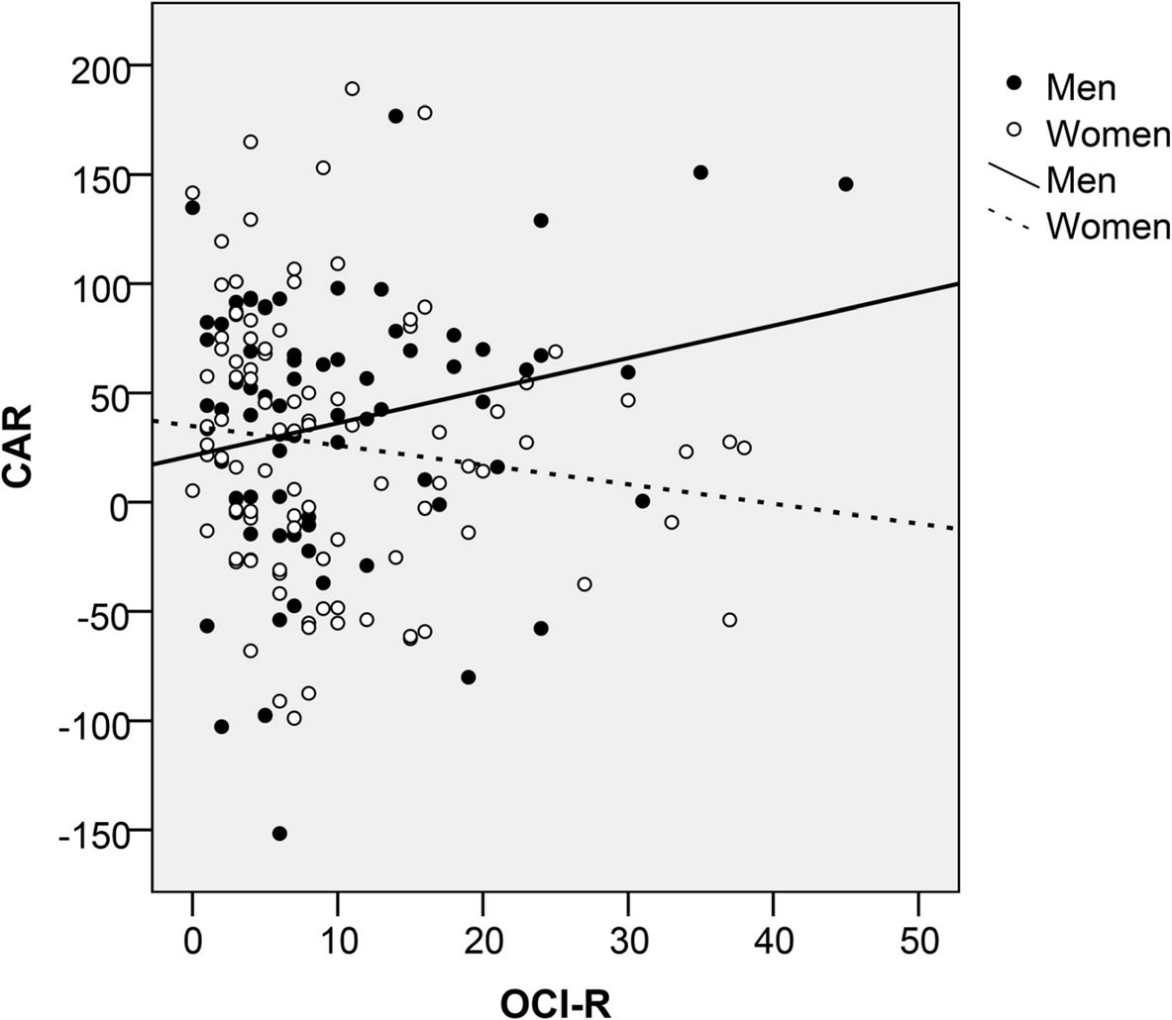
Scatterplot of the relationship between obsessive-compulsive symptoms and the cortisol awakening response by sex

Results from the three different multiple regression analysis considering OC symptoms (OCI-R total score) as the main independent variable and HPA axis measurements as dependent variables, are shown in Table 4. We found a female sex by OC symptoms significant interaction (standardized beta = - 0.322; p=0.023), indicating that the relationship between OC symptoms and the CAR differs between men and women. This interaction has been depicted in Fig. 1, as there is a positive association between OC symptoms and the CAR in men, whereas a negative association is found in women. No associations were found between OCI-R scores and other HPA axis measures (diurnal cortisol slope, DSTR). Trait anxiety was associated with an increased DSTR (Table 3).

**Table 4:** Results of the multiple linear regression analyses exploring the relationship between obsessive-compulsive symptoms and HPA axis measures

	CAR		Diurnal cortisol slope		DSTR	
	Beta	*p*	Beta	*p*	Beta	*p*
Female gender	0.115	0.306	− 0.085	0.302	0.070	0.388
Age	− 0.316	0.001	− 0.040	0.698	0.146	0.149
BMI	0.029	0.756	− 0.074	0.445	− 0.090	0.346
Smoking (cig/day)	0.054	0.489	0.028	0.726	− 0.020	0.801
OCI-R total score	0.225	0.062	0.060	0.507	− 0.123	0.167
HRSRS stress score	0.023	0.777	0.082	0.348	− 0.027	0.752
STAI-Trait Anxiety	0.037	0.742	0.134	0.251	0.264	0.022
STAI-State Anxiety	− 0.071	0.469	− 0.147	0.151	− 0.120	0.234
Female gender × OCI-R interaction	− 0.322	0.023				

Abbreviations: *CAR* cortisol awakening response, *DSTR* dexamethasone suppression test ratio, *BMI* body mass index, *OCI-R* Obsessive-Compulsive Inventory—Revised, *HRSRS* Holmes-Rahe Social Readjustment Scale, *STAI* State-Trait Anxiety Inventory

When repeating the multiple linear regression analyses for each OCI-R subscore, a female sex by OC symptoms from the obsessing dimension was found in the analyses exploring the effect on the CAR (standardized beta = − 0.252, *p* = 0.032), which means that women with more obsessions show a more blunted CAR response. In the analyses regarding the diurnal cortisol slope, OC symptoms from the ordering dimension were associated with a more flattened diurnal cortisol slope (standardized beta = 0.365, *p* = 0.006), and a significant female sex by OC ordering symptoms significant interaction was also found (standardized beta = − 0.370, *p* = 0.019). These results indicate that men with ordering symptoms show a more blunted diurnal cortisol slope whereas women show a more negative slope. There were no significant associations between OCI-R dimensions nor female sex by OCI-R dimensions interactions in those equations for the DSTR.

When repeating the analyses excluding the two women taking oral contraceptive pills, the results did not change.

## Discussion

Our study suggests that obsessive-compulsive symptoms in healthy individuals are associated with subtle abnormalities in HPA axis measures and this association is moderated by sex. A blunted CAR was associated with the severity of both overall OC symptoms and obsessing symptoms in women. A more flattened diurnal cortisol slope was associated with ordering symptoms in men. All these findings were adjusted for stressful life events and trait and state anxiety, suggesting an independent effect of OC symptoms on HPA axis activity.

There is little information in the scientific literature regarding the relationship among OC symptoms and HPA axis status. In fact, to our knowledge there is only one study that has explored the differences in CAR or diurnal cortisol slope in patients with OCD [6]. In that study from our group, we did not find significant differences between OCD patients and healthy individuals in CAR although OCD patients with comorbid major depression had a more flattened cortisol diurnal slope. Few studies have explored the HPA axis sensitivity of the negative feedback with the classical DST test (administration of 1 mg of dexamethasone and assessing cortisol suppression in plasma), suggesting that OCD patients show less suppression to dexamethasone than healthy controls [40], particularly if they have comorbid major depression [41, 42]. However, in recent studies from our group using a very low dose of dexamethasone and assessing the DSTR in saliva, no differences were found between OCD and healthy controls nor between OCD patients with or without comorbid depression [6]. In that study a positive association between trait anxiety and DSTR was found, which is in accordance with our results in the current study, suggesting that trait anxiety is more relevant for the DST than OC symptoms.

Although no previous studies have explored whether there are sex differences in the relationship between OC symptoms and HPA axis measures in healthy individuals, some studies have explored whether there are sex differences in the association between neuroticism and HPA axis indices. In a previous study that included undergraduated students and that assessed neuroticism with the NEO five-factor inventory, salivary cortisol at midday (between 10:30 a.m. and 2:30 p.m.) were positively associated with neuroticism in men and negatively in women [43]. In another study that included university students that were also assessed with the NEO 5-factor inventory, men had more flattened diurnal cortisol slopes but not differences in the CAR [44]. These two studies are in line with our results that suggest more flattened cortisol slopes in healthy individuals with more ordering OC symptoms. However, it is difficult to compare our results with the scientific literature because particular studies focused on OC symptoms are lacking. Clearly, further studies are needed to replicate our findings. The distinct associations between particular OC symptom dimensions and HPA axis measures might be explained by differences in the brain correlates of these symptom dimensions. As discussed in a recent review [45], several OCD studies have found that increased severity of aggressive/checking obsessions to be related to smaller gray matter volume in the temporal lobes, extending into the amygdala and insula, as well as the left orbitofrontal cortex (OFC), putamen, and right cerebellum volume. In contrast, findings related to the ordering/symmetry dimension are less clear, and have included both bigger and smaller volume of OFC, as well as bigger volume of other frontal regions such as the dorsal anterior cingular cortex and medial frontal cortex [45]. Other studies have reported reduced hippocampal volumes in OCD patients with more severe ordering and checking symptoms [46]. The hippocampus, which exerts negative feedback on the HPA axis via glucocorticoid and mineralocorticoid receptors, shows sex differences in response to stressors, with less remodeling of hippocampal CA3 dendrites in females after chronic stress [47]. The hippocampus is also thought to play a role in the cortisol awakening response, as subjects with hippocampal damage show a blunted CAR [48]. Thus, it is plausible that some findings regarding sex differences on the association between OC symptoms and HPA axis measures (e.g., blunted CAR and more prominent obsessing symptoms in women) could be explained by sex differences in neurobiological substrates or brain regions that are involved both in HPA axis regulation and the clinical expression of OC symptoms. In line with this hypothesis, in the MRI study by Ress et al. [46] which assessed the relationship between OC symptom dimensions and hippocampal volumes, greater volume reductions in the hippocampus were observed in a subset of OCD patients that reported greater obsessing and checking symptoms. Interestingly, in this study, there was a significant main effect of sex on global hippocampal volume, which suggests that sex is a moderating variable in the relationship between OC symptoms and hippocampal volumes. If this is the case, it is plausible that there might also exist sex differences in the relationship between the CAR and obsessing symptoms, because of the important role of the hippocampus on both the regulation of the negative feedback of the HPA axis and the CAR.

Some methodological decisions and limitations of our study need to be discussed. We aimed to study the relationship between OC symptoms and HPA axis measures in a non-clinical sample. This decision limits the generalizability of the findings to individuals with OCD. However, it allows to test the association between less severe OC symptoms and subtle HPA axis abnormalities that are not influenced by the severity of a clinical diagnosis, comorbid conditions (e.g., major depression), or treatments. This “less biased”’ approach might help to better explore whether a dysregulation of the HPA axis is contributing to OC symptoms. We did only assess the CAR over one day. As dexamethasone was administered at 11:00 p.m., we dismissed the possibility of collecting further CAR sampling the next day. Although participants were instructed to collect the saliva samples at home at the specific times, the timing of the sampling was not verified with objective methods (e.g., electronic monitoring systems). Inaccurate sample timing can bias CAR estimates [13]. Therefore, it is possible that differences in the accuracy of sampling times might be driven by obsessive-compulsive symptoms (e.g., more accurate sampling timing in people with more obsessive-compulsive symptoms). As already explained in previous studies from our group [6, 49], we used a very low dose of dexamethasone (0.25 mg) because salivary cortisol presents more profound suppression than does plasma cortisol and because we aimed to explore the DSTR as a continuous measure (ratio), and the use of higher dexamethasone doses would not have allowed us to detect subtle alterations in HPA axis regulation. The cross-sectional design of our study prevents us from inferring causality in the association between OC symptoms and HPA axis measures. Some exploratory analyses were not corrected for multiple testing (e.g., associations between distinct OC symptom dimensions and different HPA axis measures) [50]. However, it is important to underscore that these results on OC symptom dimensions are exploratory and that they need to be confirmed in further confirmatory studies.

### Perspectives and significance

Although we did not find sex differences in OC symptoms, state/trait anxiety or HPA axis measures in healthy individuals, we found a different association between HPA axis activity and OC symptoms in women and men. In summary, our study suggests that sex is a moderator of the relationship between OC symptoms and HPA axis functionality, as women with more obsessions showed a more blunted CAR response. Future studies need to replicate our findings in clinical populations including OCD patients in order to know whether these sex differences are also present in a more severe phenotype. Our study suggests that distinct OC symptom dimensions might show different associations with HPA axis measures, and points out to the possibility that these differences could be secondary to potential alterations in neural substrates that are involved in the regulation of the HPA axis and the clinical expression of OC symptoms. Although this is a speculative issue, future research is needed to shed light on this issue. In addition, psychoneuroendocrinological studies exploring the association between OC or anxiety symptoms with HPA axis activity need to control for potential sex differences.

## Data Availability

Additional data regarding the current study are available from the corresponding author on reasonable request.
